# Therapeutic Effect of a Novel Oxazolidinone, DA-7867, in BALB/c Mice Infected with *Nocardia brasiliensis*

**DOI:** 10.1371/journal.pntd.0000289

**Published:** 2008-09-10

**Authors:** Lucio Vera-Cabrera, Alejandra Daw-Garza, Salvador Said-Fernández, Hector Gerardo Lozano-Garza, Noemi Waksman de Torres, Norma Cavazos Rocha, Jorge Ocampo-Candiani, Sung-Hak Choi, Oliverio Welsh

**Affiliations:** 1 Servicio de Dermatología, Hospital Universitario “José E. González”, Monterrey, Nuevo León, México; 2 Centro de Investigación Biomédica del Noreste, IMSS, Monterrey, Nuevo León, México; 3 Departamento de Química Analítica Facultad de Medicina, U.A.N.L., Monterrey, Nuevo León, México; 4 Research Laboratory, Dong-A Pharmaceutical Co., Ltd., Yongin, and College of Pharmacy, Sungkyunkwan University, Suwon, Korea; New York Blood Center, United States of America

## Abstract

**Background:**

Mycetoma is a chronic infectious disease of tropical and subtropical countries. It is produced by true fungi and actinobacteria. In México, *Nocardia brasiliensis* is the main causative agent of mycetoma, producing about 86% of the cases; the gold standard for the therapy of mycetoma by *N. brasiliensis* is the use of sulfonamides which give a 70% cure rate. The addition of amikacin to this regime increases to 95% the cure rate; however, the patients have to be monitored for creatinine clearance and audiometry studies because of the potential development of side effects. Because of that it is important to search for new active compounds. In the present work, we evaluated the in vivo effect of DA-7867, an experimental oxazolidinone, on the development of experimental mycetomas by *N. brasiliensis* in BALB/c mice.

**Methodology/Principal Findings:**

In order to determine the optimal dose utilized to apply to the animals, we first determined by HPLC the plasma levels using several concentrations of the compounds. Based on these results, we used 10 and 25 mg/kg subcutaneously every 24 hr; DA-7867 was also supplied in the drinking water at a calculated dose of 25 mg/kg. As a control we utilized linezolid at 25 mg/kg, a compound active in murine and human infections, three times a day. The mice were infected in the right footpad with a young culture of *N. brasiliensis* HUJEG-1, and one week later we started the application of the antimicrobials for six more weeks. After that we compared the development of lesions in the groups injected with saline solution or with the antimicrobials; the results were analyzed by the variance ANOVA test. DA-7867 was able to reduce the production of lesions at 25 mg/kg, when given either subcutaneously or in the drinking water.

**Conclusions/Significance:**

The experimental oxazolidinone DA-7867 is active in vivo against *N. brasiliensis*, which opens the possibility of using this drug once it is accepted for human application. Since oxazolidinones seem to be active against a wide spectrum of actinobacteria, it is possible they could be used in human cases of mycetoma by other actinomycetales, such as *Streptomyces somaliensis,* highly prevalent in Sudan, or *Actinomadura madurae* and *A. pelletieri*, which are commonly observed in Africa and India.

## Author Summary

Actinomycetoma is an infectious disease of tropical and subtropical regions produced by actinobacteria of the genera *Nocardia*, *Streptomyces*, and *Actinomadura*. Therapeutic alternatives are scarce and include trimethoprim-sulfamethoxazole, diaminodiphenylsulfone, amoxicillin-clavulanate, imipenem, and amikacin. Oxazolidinones are a new class of antimicrobials with a completely different cellular target; the first compound in the market, linezolid, was introduced in the year 2000. It is active against many species of *Nocardia* and other aerobic actinomycetes; however, the long-term application in human subjects produces side effects including peripheral neuropathy and mielossupression. Therefore, it is important to screen other oxazolidinones with higher activity and less toxicity. In the present work, we tested DA-7867, a new oxazolidinone, in an experimental mouse model. The drug is active in vivo and decreases the production of lesions using only one dose a day in contrast to linezolid, which needs to be injected three times a day. Although it was tested on *N. brasiliensis*, it can possibly be active (once it is accepted for its use in humans) against *Actinomadura spp* and *Streptomyces spp*, which are frequently found in places of Africa and India where actinomycetoma is also an important consult in dermatology.

## Introduction

Mycetoma is a multi-etiological subcutaneous infection caused by true fungi and aerobic actinomycetes observed in many tropical and sub-tropical countries. In Mexico, 98% of the total cases are produced by actinomycetes and about 86% are produced by *N. brasiliensis* [1]. Many antimicrobials have been used in the therapy of actinomycetoma, including sulfonamides (DDS, sulfamethoxypyridazine, and sulfadoxine), streptomycin in combination with dapsone or with trimethoprim-sulfamethoxazole (SXT), minocycline, imipenem, amoxicillin-calvulanic acid, etc. [2]. The highest cure rates (70%) have been obtained with the use of sulfamethoxazole-trimethoprim (SXT). In our dermatology clinic we have added amikacin to the SXT combination to treat severe cases of mycetoma or those cases involving subjacent organs, obtaining a cure rate of about 95% in a series of 52 patients [3]. However, in some cases, the use of these antimicrobials carries the risk of side effects or bacterial resistance.

Oxazolidinones are recently developed antimicrobials that inhibit protein synthesis in a site not targeted by other antimicrobials [4]. Resistance by mutations or the presence of a resistance determinant has rarely been reported. The first of these compounds to be approved by the Food and Drug Administration (FDA), linezolid, has been observed to be active in vitro against several gram-positive actinobacteria including *Nocardia*, *Actinomadura*, and *Mycobacterium tuberculosis* [5],[6],[7]. Linezolid has been successfully tested by us in an experimental model of infection with *N. brasiliensis* in BALB/c mice, and this activity has been confirmed in human infections [8],[9]. However, in long term applications, linezolid produces side effects, such as myelosuppression and peripheral neuropathy [10]. Therefore it is important to look for more powerful and less toxic oxazolidinones. Recently, an experimental oxazolidinone, DA-7867 ((S)-[N-3-(4-[2-(1-methyl-5-tetrazolyl)-pyridin-5-yl]-3-fluorophenyl)-2-oxo-5-oxazolidinyl] methyl acetamide) has been observed to be highly active in vitro against *N. brasiliensis* isolates [5], even at much lower concentrations than linezolid, which makes this drug an excellent candidate to be tested in vivo.

In the present work, we used an animal model to study the in vivo activity of this experimental compound, and compared its effectiveness with that of linezolid.

## Methods

### Microorganisms

For the animal assays we utilized *Nocardia brasiliensis* HUJEG-1 which has been utilized in previous studies [8],[11],[12]. The MIC values of this strain are 0.03 µg/ml for DA-7867, and 0.12 µg/ml for linezolid. The strain was grown on Sabouraud dextrose agar for 7 to 10 days and a suspension in 20% skim milk of each was made. These bacterial suspensions constituted our stock cultures that were kept at −70°C in cryovials until use.

### Determination of plasma levels of the antimicrobials

Several doses of each compound linezolid or DA-7867 were used to determine their plasma concentrations in BALB/c mice. Linezolid was used at 10 mg/kg, 25 mg/kg and 50 mg/kg; DA-7867 at 10 mg/kg and 25 mg/kg. Eight-to-twelve week-old female BALB/c mice were injected subcutaneously with the antimicrobials. For each dose tested, 27 mice were utilized; 24 were injected with the selected dose and 3 mice were not injected to represent time 0. Next, 500 µl blood samples were taken from the infraorbitary sinus of each mouse, which previously had undergone general anesthesia with ethylic ether. The samples were taken from groups of 3 mice each at the following time intervals: 0 min, 20 min, 40 min, 1 hr, 2 h, 4 h, 6 h, 8 h and 10 h.

After sample collection, the plastic tubes containing the blood were centrifuged and the plasma separated and frozen at −70°C. Plasma concentrations were determined by using by using a previously validated HPLC (High Performance Liquid Chromatography) method as follows [13]. The serum protein was precipitated by combining 50 µL of sample with 150 µl of acetonitrile. The mixture was vortexed for 10 sec and centrifuged for 5 min at 5304×g, and the supernatant was filtered through 0.45 µm Nylon filters (Waters). Filtrates were received into 150 µL inserts for chromatographic analysis. The chromatographic separation of antimicrobials was achieved using a Waters 2690 Alliance liquid chromatograph with diode array detector 996 (DAD) and fluorescence detector 474. An Atlantis dC18 column 150 mm×4.6 mm I.D., with 5 µm particle size (Waters) was used. Column temperature was maintained at 30°C. Samples were eluted with a mobile phase consisting of 0.1% trichloroacetic acid (solvent A) and acetonitrile (solvent B); a gradient program was utilized for the elution. In the case of DA-7867, in order to reduce the retention time of this analyte, this program was modified with an increase in the initial proportion of acetonitrile. The flow rate of the mobile phase was 1.0 mL/min and the injection volume was 10 µL. The DAD wavelength was set at 254 nm for linezolid; for DA-7867 the fluorescence intensities were measured at an excitation wavelength of 292 nm and an emission wavelength of 408 nm.

### Inoculum preparation for the animal assay

*N. brasiliensis* HUJEG-1 was cultured in Sabouraud agar for 1 week, and then cultured in Brain-Heart-Infusion broth at a temperature of 37°C in a shaker at 110 RPM for 72 hrs. The bacterial mass was then separated by centrifugation, and washed 4-times with saline solution. After grinding in a Potter-Evelham device the suspension was left to sediment and the bacterial mass was washed one more time with saline solution and adjusted to 20 mg (wet weight) of *N. brasiliensis* per 50 µL of saline solution.

### Experimental infection

Eight-to-twelve week old female BALB/c mice were inoculated with 20 mg of *Nocardia brasiliensis* in the right hind foot-pad. Seven days later the therapeutic assay was started.

### Therapeutic assays

Groups of 15 mice each were used. One group was injected subcutaneously in the back with 0.1 ml pyrogen-free saline solution; the rest were treated with DA-7867 at a dose of 10 mg/kg and 25 mg/kg daily, and linezolid at a dose of 25 mg/kg three times a day. The drugs were administered subcutaneously on the back of each mouse during a four week period.

Since it is more comfortable for the patient to take the compounds orally, we studied this possibility by giving DA-7867 in this way. However, since it was a long term experiment (the antimicrobial had to be given five times a week during four weeks ) and to avoid a possible esophageal ripping due to the insertion of the oral cateter, we decided to give it in the drinking water. DA-7867 was suspended in 10% hydroxypropylcellulose and given in the drinking water at a dose of 25 mg/kg. The amount of compound was calculated according to the amount of water drank during the day per each animal. Since the amount of antimicrobial taken by the animals was not completely controlled we determined the levels in the plasma of the animals by the HPLC method described above at 0, 3, 6, 9, and 12 h after they started to drink a new bottle.

### Evaluation of the therapeutic assays

The lesions in the foot pad were scored based on the degree of extension as published previously [8], with scores ranging from 0 for those presenting absolutely no lesions or inflammation to 4+ for the animals presenting severe lesions extending above the metatarsal bones. Differences among the therapeutic group were analyzed with an ANOVA test and confirmed with the Dunnet test.

The study was approved by the Comité Local de Investigación en Salud No. 1908, Centro de Investigación Biomédica del Noreste, IMSS, and the animal handling was done according to our institutions' guidelines.

## Results

### Antimicrobial plasma levels

**Figure 1:**
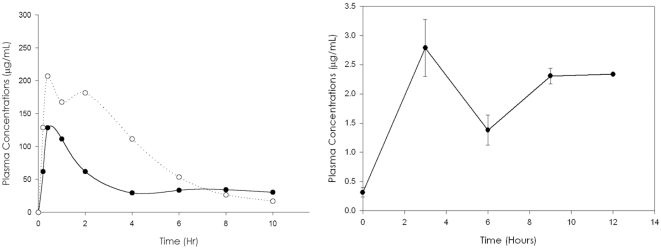
Mouse plasma levels of DA-7867 given subcutaneously (left) and in the drinking water (right). DA-7867 was given s.c. at 10 (•) and 25 (○) mg/kg. In the right picture we show the levels produced after giving the compound at a dose of 25 mg/kg in the drinking water. Each point represents the mean of three animals.

The concentrations of linezolid in plasma of BALB/c mice has been published before [12]. At 25 mg/kg it keeps concentrations above the MIC value (0.12 µg/ml) for more than 4 hr, with a *Cmax* of 50 µg/ml. On the other hand, DA-7867 maintains concentrations over the MIC (0.03 µg/ml) even at 10 h at 10 and 25 mg/kg, with a *Cmax* of about 200 µg/ml at 25 mg/kg (Fig 1). The plasma levels found in rats are different as reported by Bae et al [14].

When given in the drinking water plasma concentrations of the compound were over the MIC the 12 h, with a *Cmax* of 2.8 µg/ml at 3 h after start drinking (Fig 1).

### Experimental model of actinomycetoma

This actinomycetoma model has been described in a former publications [8],[11],[12]. In brief, inoculation of 20 mg wet weight of *N. brasiliensis* culture, produced a highly inflammatory response 5 days post inoculation. After this point footpad thickness decreases and at the end of 4 weeks, there is the installation development of real mycetoma lesions. If left for months, giant mycetoma lesions develop and remain localized. To interpret the therapeutic effect of the various compounds, we decided to treat the animals for 4 weeks by which time the lesions are installed, and they have reached the equivalent size of a human mycetoma.

### Therapeutic effect of DA-7867

**Figure 2:**
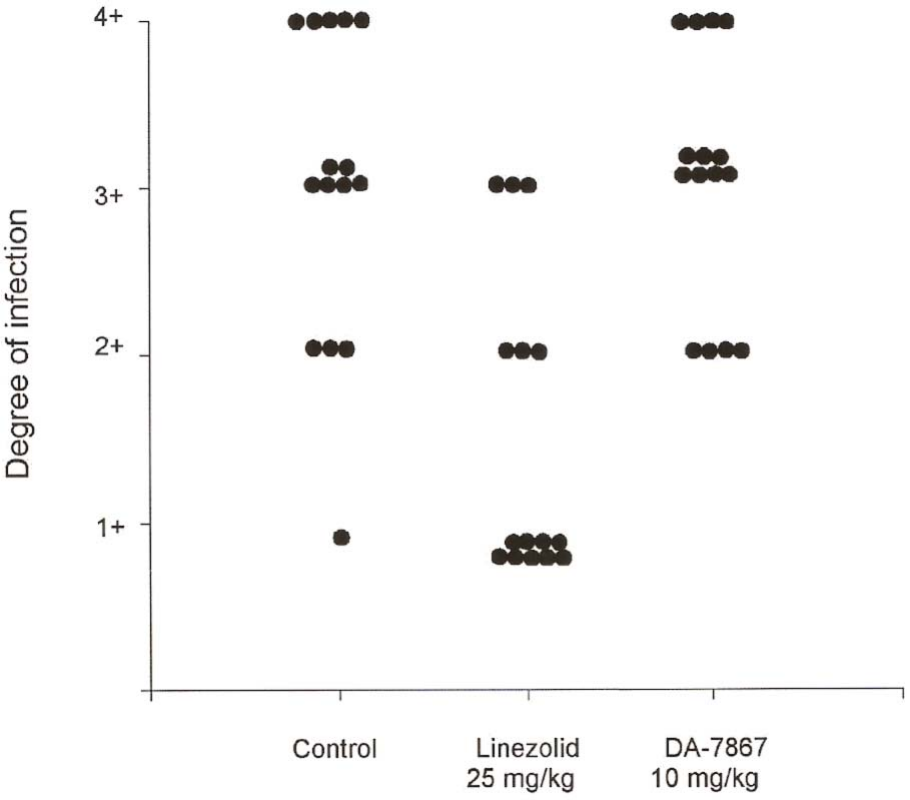
Effect of the application of saline solution, linezolid, or DA-7867 (at 10 mg/kg per day) on the development of lesions by *N. brasiliensis* on the mouse footpad. Statistically significant (p<0.005) differences from the control animals were observed only in the group treated with linezolid.

**Figure 3:**
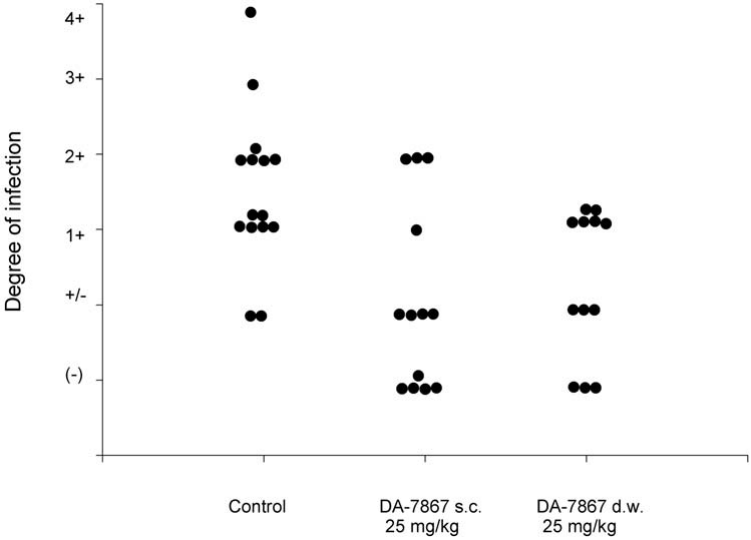
Effect of the application of DA-7867 at 25 mg/kg, subcutaneously or in the drinking water, in the development of experimental lesions by *N. brasiliensis*. Differences in the number of animals were considered for the variance statistical analysis. In both cases statistically significant differences with respect to the control were observed (p = 0.002 in both cases).

In Fig. 2, we observe the effect of DA-7867 at 10 mg/kg every 24 hr. At this first dose, DA-7867 had no effect on the evolution of the mycetoma lesions, and this therapeutic group was undistinguishable from the control. When increasing the dose to 25 mg/kg a clear effect on the production of mycetomatous lesions was observed (Fig 3). When the results were analyzed with the one-way ANOVA test, only the treatments with DA-7867 at 25 mg/kg and linezolid were statistically significant (p = 0.002). Equal results were obtained when analyzing the mean values with the Dunnet test.

In the group of animals were DA-7867 was given in the drinking water at 25 mg/kg (Fig 3), we observed a comparable effect to that obtained when the antimicrobial was applied subcutaneously at the same concentration, observing also a significant statistical difference with the ANOVA (p = 0.002) and Dunnet tests when comparing with the group receiving saline.

## Discussion

Currently there are not many alternatives for the therapy of actinomycetoma by *N. brasiliensis*. The most common antimicrobials utilized, SXT and the combination SXT-amikacin are not 100% effective, and it is known that these compounds can induce side effects such as Stevens-Johnson syndrome, hypacusia, and nephrotoxicity. Since antimicrobials have to be taken for long periods of time, adherence to treatment is very important to avoid the appearance of resistance. However most of the patients are poor, ill-educated and live in rural areas far from hospital and when they come back to the consult it is because of remission of the disease. Therefore, more potent and less toxic compounds are needed.

Quinolones such as gatifloxacin, moxifloxacin and garenoxacin, as well as linezolid, a compound of the class of the oxazolidinones, are among the new antimicrobials tested against *N. brasiliensis* which are active in vitro against the 100% of the isolates tested [5],[8]. Of them only gatifloxacin and linezolid have been tested in the mouse model of actinomycetoma by *N. brasiliensis* with positive results [8],[12]; unfortunately, gatifloxacin has been withdrawn from the market because of the report of the production of disglycemia [15]. Its return to the pharmaceutical shelves will depend very much on the results of the clinical trials with patients with tuberculosis, where it seems to be as active as ethambutol [16].

On the other hand, linezolid has been used to treat successfully some cases of subcutaneous nocardiosis; however after several months of use neurological and haematological side effects were observed in some patients [9]. In our experience, we treated a patient presenting a case of subcutaneous nocardiosis of the leg, with dissemination to the abdomen and formation of large retro-psoas abscesses. The infection was produced by an isolate clinically resistant to amikacin, SXT, and amoxicillin-clavulanic acid; the patient was successfully treated with 600 mg daily of linezolid for 3 months without the production of side effects. This encouraged us to look for new drugs for resistant cases of actinomycetoma. In previous in vitro studies we observed that *Nocardia spp* and *Actinomadura madurae* isolates are susceptible to antimicrobials not previously used in patients with mycetoma, including new oxazolidinones [4]. One of them DA-7867, was observed to be even more active than linezolid and amikacin. These in vitro results made them excellent candidates for in vivo experimentation.

In a previous work, we have observed that plasma levels of linezolid in BALB/c mice at a dose of 50 mg/kg remains for about 4 hr over the MIC value, with a *Cmax* of of 70 µg/ml [12]. DA-7867 reaches much higher concentrations in plasma with a *Cmax* of 200 µg/ml at 25 mg/kg, maintaining levels above the MIC (0.03 µg/ml) for more than 10 hr. Because of the high levels observed in plasma we decided to use it in a low dose (10 mg/kg), and only once a day; however under this conditions we did not observe any clinical differences compared to the control. When using a higher dose (25 mg/kg, daily), we could observe a therapeutic effect, comparable to that of linezolid applied three times daily. This is perhaps due to the exquisite susceptibility of *N. brasiliensis* to this drug (MIC = 0.03 µg/ml), compared to 0.12 µg/ml of linezolid. Importantly, when it was given in the drinking water at a calculated dose of 25 mg/kg, even if the amount of compound taken was not controlled we observe a comparable effect to the drug given subcutaneously. It is important to remark that appearance of the animals was excellent, since we did not had to manipulate them at all, compared to the groups of animals that had to be injected subcutaneously several times a day. These results open the possibility to give DA-7867 orally instead than subcutaneously, which is more comfortable for the patient. This is important since adherence to the treatment is essential for successful long term therapeutic schemes.

One of the most important factors which determine the use of experimental drugs in humans is their poor solubility in water. Recently, Dong-A-Pharm has developed a pro-drug DA-7218, a new oxazolidinone, which in the body is dephosphorylated to the active compound DA-7257 [14]. In contrast to linezolid and DA-7867, DA-7218 is highly soluble in water (greater than 150 mg/ml at pH 7.0) and it is quite active against *N. brasiliensis* in vivo, even at 5 mg/kg. It is important to mention that therapy with DA-7867 and DA-7218 produced animals with no lesions or slight inflammation (scored in Fig. 3 as neg or +/−); experimental therapy with other compounds such as linezolid, or amoxicillin-clavulanate only produced decrease of the lesions, but without curing the infection completely [8]. It is possible that this new oxazolidinones have a more potent bactericidal effect, which will be beneficial for the patients, although we still will have to wait to test them until they are approved for human use.

## Supporting Information

Alternative Language Abstract S1 Translation of the Abstract into Spanish by Lucio Vera-Cabrera
(0.03 MB DOC)
