# Supplementary material for: Therapeutic Effect of a Novel Oxazolidinone, DA-7867, in BALB/c Mice Infected with Nocardia brasiliensis
Source: PLoS Negl Trop Dis. 2008 Sep 10;2(9):e289. doi: 10.1371/journal.pntd.0000289 (PMC2553479; doi:10.1371/journal.pntd.0000289)
Supplement: Alternative Language Abstract S1 — Translation of the Abstract into Spanish by Lucio Vera-Cabrera (0.03 MB DOC) [file pntd.0000289.s001.doc]

**RESUMEN**

Introduccion: El micetoma es una enfermedad infecciosa crónica propia de países tropicales y sub-tropicales. Es producida por hongos verdaderos y actinobacterias. En Mexico, *Nocardia brasiliensis* es el agente causal mas comúnmente aislado y produce alrededor del 86% de los casos; el estándar de oro de la terapia del micetoma por *N. brasiliensis* es el uso de sulfonamidas que es efectivo aproximadamente en el 70% de los casos. La adición de amikacina a este esquema terapéutico incrementa al 95% la tasa de curación, sin embargo a los pacientes se les tiene que realizar periódicamente estudios de eliminación de creatinina y audiometrías por si se desarrollan efectos secundarios. Debido a esto es importante analizar nuevos compuestos. En el presente trabajo, evaluamos el efecto in vivo de DA-7867, una oxazolidinona experimental, en el desarrollo de lesiones experimentales por *N. brasiliensis* en ratones BALB/c.

Metodologia/hallazgos mas importantes: Para determinar la dosis optima de los medicamentos para aplicar a los animales, primero determinamos por HPLC los niveles en plasma utilizando varias concentraciones de los compuestos. Basados en estos resultados, utilizamos 10 y 25 mg/kg del compuesto aplicados subcutáneamente cada 24 hr; DA-7867 fue también dado en el agua de tomar a una dosis calculada de 25 mg/kg. Como control utilizamos linezolid a 25 mg/kg tres veces al día, un compuesto que ha demostrado ser activo en infecciones experimentales en ratones, así como en humanos. Los animales fueron infectados en el cojinete plantar derecho con un cultivo joven de *N. brasiliensis* HUJEG-1 y una semana después se empezaron a aplicar los antimicrobianos por un periodo de seis semanas. Al término de este tiempo, comparamos el desarrollo de lesiones en los grupos inyectados con solución salina y los tratados con los antimicrobianos; los resultados fueron analizados con la prueba de varianza de Anova. Observamos que DA-7867 a 25 mg/kg, tanto aplicado subcutáneamente o dado en el agua de tomar fue capaz de reducir la producción de lesiones.

Conclusiones, significancia: La oxazolidinona experimental DA-7867 es activa in vivo contra *N. brasiliensis* lo que abre la posibilidad de usarla una vez autorizado su uso en humanos. Ya que las oxazolidinonas parecen ser activas contra un amplio espectro de actinobacterias, es posible su uso en casos humanos de micetoma producidos por otros actinomicetos como *Streptomyces somaliensis,* comúnmente aislado en Sudán, y *Actinomadura madurae* o *A. pelletieri*, que son comúnmente observados en África e India.
